# High rates of International Code violations: a cross-sectional study in a region of Canada with low breastfeeding rates

**DOI:** 10.1186/s13104-024-06725-8

**Published:** 2024-03-12

**Authors:** Susan Barry, Hannah Buckle, Leigh Anne Allwood Newhook, Barbara Roebothan, Brittany Howell, Heather Gates, Laurie K. Twells

**Affiliations:** 1 Division of Population Health and Applied Health Sciences, Faculty of Medicine| Memorial University of Newfoundland, A1B 3V6 St. John’s, Newfoundland and Labrador, Canada; 2 Labrador-Grenfell Health, Happy Valley-Goose Bay, NL Canada; 3 Faculty of Medicine, Memorial University, St. John’s, Newfoundland and Labrador, Canada; 4 Baby-Friendly Council of NL, Baby-Friendly Newfoundland and Labrador, St. John’s, Newfoundland and Labrador, Canada

**Keywords:** International Code of Marketing of Breast-Milk Substitutes, Violations, Breastfeeding, Canada

## Abstract

**Background:**

Exposure to marketing and promotion of commercial milk formula is associated with an increased likelihood of formula-feeding. In 1981, the International Code (IC) of Marketing of Breastmilk Substitutes was adopted by the 34th World Health Assembly to restrict the promotion, marketing and advertising of commercial milk formula and protect breastfeeding.

**Research Aim:**

The current study examines mothers’ exposure to violations of the IC in Newfoundland and Labrador, a province of Canada with low breastfeeding rates.

**Methods:**

A cross-sectional online survey measured exposure to IC violations (e.g., marketing, advertising and promotion of commercial milk formula) by mothers of infants less than two years old (*n* = 119). Data were collected on type, frequency, and location of violation.

**Results:**

Most participants (87%, *n* = 104/119) reported exposure to at least one IC violation. Of this group (*n* = 104): 94% received coupons or discount codes for the purchase of commercial milk formula; 88% received free samples of commercial milk formula from manufacturers, and 79% were contacted directly by commercial milk formula companies via email, text message, mail or phone for advertising purposes. One-third (*n* = 28/104, 27%) observed commercial milk formula promotional materials in health care facilities. The most frequent locations were violations occurred were doctors’ offices (79%), supermarkets(75%), and pharmacies (71%).

**Conclusion:**

The majority of mothers of young infants were exposed to violations of the IC involving the marketing, advertising and promotion of commercial milk formula. Companies producing commercial milk formula reached out directly to new mothers to offer unsolicited promotions and free samples of commercial milk formula.

**Supplementary Information:**

The online version contains supplementary material available at 10.1186/s13104-024-06725-8.

## Background

In 1981, concerns about the marketing practices of the infant formula industry and a global decline in breastfeeding led to the 34th World Health Assembly (WHA) calling for the regulation of the infant formula industry. To protect against breastfeeding, the World Health Organization (WHO) adopted the International Code of Marketing of Breast-Milk Substitutes (IC) to reduce the unethical marketing practices associated with infant formula [1]. The IC includes eleven articles (Supplementary Table) and subsequent WHA resolutions that recommended manufacturers and distributors of infant formula stop all forms of inappropriate promotion of infant formula, and that governments take action to legislate, implement, and monitor the resolutions of the IC and WHA [1]. As of 2022, according to a global report by the WHO, the United Nations International Children’s Emergency Fund (UNICEF) and the International Baby Food Action Network (IBFAN), 74% of countries in regions of Africa, the Eastern Mediterranean and South East Asia have enacted some legal measures with provisions to implement and monitor adherence to the IC. These legal provisions include defining an IC violation, identifying groups or organizations to monitor compliance with the IC and ensuring those who are responsible for compliance are not influenced by manufacturers of infant formula.

Within the remaining 35% of countries, Canada and the United States have no legal measures in place [2].

Although the WHO designated the IC as a “minimum requirement” for member states, individual countries need legislation to support the tenets of the IC in order to hold organizations responsible for non-adherence [3]. An increase in breastfeeding rates has been observed in countries that have adopted legislation supporting the IC [3–4]. Although Canada approves the IC, there is no legislation in place to ensure its implementation and compliance [2].

The reasons for the global decline in breastfeeding are multifactorial [5]. Exposure to commercial milk formula is one significant factor associated with an increased likelihood of formula feeding [3]. The extent of the marketing, advertising and promotion of commercial milk formula by manufacturers is demonstrated by the increase in global production, marketing, and sales of commercial milk formula, the value of which has grown from US$1.5 billion in 1978 to US$55.6 billion in 2019 [6].

In 2022, the WHO and UNICEF published the results of a two-year multicountry study (2019–2021), the overall aim of which was to examine the formula milk marketing landscape in eight countries. Data were collected in Bangladesh, China, Mexico, Morocco, Nigeria, South Africa, the United Kingdom and Vietnam, countries with a range of exclusive breastfeeding rates (21–65%). These countries were chosen as they were representative of their regions, income levels and rates of exclusive breastfeeding. This study was novel in design, as it used nine data collection methods and applied consumer-focused methods and frameworks in the collection and analysis of the data. Study participants included pregnant women, mothers of infants and young children, and health care professionals and marketing executives. Surveys, focus groups, ethnographies and in-depth interviews were conducted. The majority of mothers in the study reported exposures to formula milk marketing from several sources, including TV, social media, and in supermarkets, hospitals, clinics and print. Women in all eight countries reported receiving free samples of infant formula. The authors of this report suggested that mothers’ exposure to commercial milk formula disrupted informed decision-making and undermined breastfeeding [7].

In another study by Lutter et al., the authors summarized studies of IC violations in eight countries between 2016 and 2020 that were published by NetCode (i.e., Network for Global Monitoring and Support for Implementation of the IC and Subsequent Resolution). Among the 389 retail stores and pharmacies surveyed, promotions for infant formula were observed in 63%, and among the sample of 3124 pregnant women and mothers of young children, 64% reported viewing the promotion of products covered by the IC, most of which (62%) were seen outside health care facilities [8].

In 2007, the authors of a Canada-wide hospital-based study reported 24% of breastfeeding mothers were given free samples of infant formula before discharge, an IC violation [9]. In 2006–2007, the Public Health Agency of Canada (PHAC) conducted a national survey of women’s perinatal experiences, perceptions, knowledge and practices up to and including early parenting. A random sample was recruited that was representative of mothers across Canada. One in three mothers (36%) reported receiving free samples of infant formula from healthcare professionals [10]. Over the last decade, limited research has been conducted in Canada on mothers’ exposure to industry violations of the IC either nationally, provincially or at the territorial level. The aim of the current study was to investigate mothers’ exposure to violations of the IC in one province of Canada.

## Methods

### Research design

A cross-sectional online survey on the exposure to violations of the IC was conducted on a sample of mothers aged 19 years or older living in the Eastern region of Newfoundland and Labrador (NL). The Provincial Health Research Ethics Board approved this study (HREB#2018.158).

### Setting and relevant context

The population of NL is 530,128, with an average of 4000 births a year [11]. The breastfeeding initiation and six-month exclusive breastfeeding (EBF) rates in NL compared to those in Canada are 72% versus 91% and 13% versus 35%, respectively [12]. The NL population has the lowest provincial breastfeeding rate in Canada [13].

### Sample and recruitment

Mothers aged 19 years of age and older living in the Eastern Region of NL who delivered at least one healthy full-term (39 + weeks) infant under the age of two at the time of the study were eligible. Recruitment was conducted through email, posters, and online posts in breastfeeding, infant formula-feeding social media groups, healthcare facilities, and family resource centers. Recruitment messaging explicitly welcomed mothers regardless of the chosen feeding method and was representative of all socio-economic groups. Participants were self-screened for eligibility at the start of the survey.

### Measurement and data collection

Data on maternal socio-demographics (i.e., age, marital status, education, household income, resident community size, employment), infant feeding methods, and exposure to IC violations were collected. The self-reported infant feeding modes included exclusive breastfeeding and mostly breastfeeding [14], mixed feeding (defined in the survey as a combination of breastmilk and infant formula) and exclusive infant formula feeding. The data on exposure to IC violations were collected based on the specific articles 5.1, 5.3, 5.4, 5.5, 6.3, and 7.4 (Supplementary Table). Survey respondents were asked to report on facilities or locations where commercial milk formula branding was viewed and to identify the providers of the samples. Survey questions were modified (with permission from IBFAN) from the IC Monitoring Materials “Form 1: Interview with mothers“ [15]. Each IC violation was transposed into a question (Table 1) that elicited a “yes or no” answer with a drop-down menu where applicable (e.g., when listing locations where promotions were viewed). The data were collected using SurveyMonkey over six weeks between September and October 2018.

**Table 1:** Violations from Articles 5–7 of the International Code (IC) Associated with Survey Questions*

Survey Question	Article from IC (p. 10–12)
Have you, your partner, or your infant ever been contacted directly or indirectly by a company regarding infant formula or another breast milk substitute?** (e.g., Nestle, Enfamil, Abbot (Similac)) such as an email, text message, mail, phone call, etc.?	5.1 There should be no advertising or other form of promotion to the general public of products within the scope of this Code. 5.5 Marketing personnel, in their business capacity, should not seek direct or indirect contact of any kind with pregnant women or with mothers of infants and young children.
Have you, your partner, or your infant ever received a coupon or discount code for infant formula or another breast milk substitute via mail, email, social media, text messaging, telephone or any other form of contact? (5.3)	5.3 In conformity with paragraphs 1 and 2 of this Article, there should be no point-of-sale advertising, giving of samples, or any other promotion device to induce sales directly to the consumer at the retail level, such as special displays, discount coupons, premiums, special sales, loss-leaders and tie-in sales, for products within the scope of this Code. This provision should not restrict the establishment of pricing policies and practices intended to provide products at lower prices on a long-term basis.
Have you, your partner, or your infant ever received free samples of infant formula or any breast milk substitute? (5.3 & 7.4)	5.3 In conformity with paragraphs 1 and 2 of this Article, there should be no point- of-sale advertising, giving of samples, or any other promotion device to induce sales directly to the consumer at the retail level, such as special displays, discount coupons, premiums, special sales, loss-leaders and tie-in sales, for products within the scope of this Code. This provision should not restrict the establishment of pricing policies and practices intended to provide products at lower prices on a long-term basis. 7.4 Samples of infant formula or other products within the scope of this Code, or of equipment or utensils for their preparation or use, should not be provided to health workers except when necessary for the purpose of professional evaluation or research at the institutional level. Health workers should not give samples of infant formula to pregnant women, mothers of infants and young children, or members of their families.
If so, which organizations or professionals have you received these samples from? Check all that apply: Infant formula or other breast milk substitute company, Supermarket, Drug store, Doctor, Nurse, Registered Dietitian, Pharmacist, Social Worker, Other health professional, Other Never received free samples	
Have you, your partner, or your infant ever received a gift from an infant formula or other breast milk substitute company or organization such as a baby bib, spoon or bottle with visible branding or logos? (5.4)	5.4 Manufacturers and distributors should not distribute to pregnant women or mothers or infants and young children any gifts of articles or utensils which may promote the use of breast-milk substitutes or bottle-feeding.
Have you or your partner ever seen any materials such as calendars, magnets, notepads, handouts, or posters bearing the brand of an infant formula or breast milk substitute company or product in a health care facility in Newfoundland and Labrador? (6.3) If so, in which type of facility have you seen these materials? Check all that apply: Doctor’s office Hospital Public health clinic Pharmacy Drug store Supermarket Other	6.3 Facilities of health care systems should not be used for the display of products within the scope of this Code, for placards or posters concerning such products, or for the distribution of material provided by a manufacturer or distributor other than that specific in Article 4.3.

*2016 resolutions now state the requirement to end inappropriate promotion of foods (not just infant formula) for infants and young children

**The term breast milk substitute refers to any food being advertised as a partial or total replacement for breastmilk, whether or not suitable for that purpose

### Data analysis

Descriptive statistics were used to report the absolute, relative frequency and proportions of the sociodemographic variables and infant feeding choices. Chi-square analysis and Fisher’s exact test (cell counts < 5) for categorical data were conducted to determine whether associations existed between socio-demographic variables, infant feeding choice and exposure to IC violations (yes or no). The statistical analysis was performed using SPSS statistical software for Windows version 23 (IBM Corporation, New York, USA).

## Results

A total of 119 participants met the eligibility criteria, with ages ranging from 21 to 42 years (mean 30.9 years, SD 3.9). The infants/children included in the study ranged from less than one month to 24 months (mean 10.0, SD 6.3). Most participants were partnered, had postsecondary education, reported an annual household gross income of $70,000 and were living in larger urban areas (Table 2). The majority of mothers (80%) were exclusively or mostly breastfeeding, while one in five were mixed feeding or exclusively formula feeding (19%). Most participants (87%, *n* = 104/119) reported being exposed to at least one IC violation. The reported violations included (i) receiving coupons or discount codes for commercial milk formula (94%, *n* = 98/104); (ii) receiving free samples of commercial milk formula (88%, *n* = 91/104); and (iii) being contacted directly by a commercial milk formula company via email, text message, mail, or phone call (79%, *n* = 82/104). Among the participants who received free commercial milk formula (88%, *n* = 91), all reported receiving it directly from commercial milk formula companies (100%). Some participants reported having received free samples from physicians (12%, *n* = 11/91), nurses (5%, *n* = 5/91), other healthcare professionals (1%, *n* = 1/91) or from where commercial milk formula was purchased (e.g., supermarkets (3%) and drugstores (1%)).

**Table 2:** Baseline characteristics of study sample (n = 119)

Characteristic*	Total sample	Exposed to Unethical Marketing Practices
	n (%)	n (%)
Total Sample	119	104 (87%)
Marital Status		
Partnered	109 (91%)	97 (94%)
Education		
Secondary or less	7 (6%)	7 (7%)
College/Trades	37 (31%)	35 (34%)
Postsecondary or above	75 (63%)	62 (60%)
Household Income		
Under $70 000	32 (27%)	30 (29%)
$70 000 or above	82 (69%)	70 (67%)
Prefer not to say	5 (4%)	4 (4%)
Community Size		
Rural (Less than 1000 residents in community)	10 (8%)	10 (10%)
Small Urban (1000 to 29,999 residents in community)	30 (25%)	26 (25%)
Medium Urban (30,000 to 99, 000 residents in community)	18 (15%)	17 (16%)
Large Urban (Greater than 100,000 residents in community	61 (51%)	51 (49%)
Employment		
Employed but currently on maternity leave	61 (51%)	54 (52%)
Full time	37 (31%)	31 (30%)
Unemployed	21 (18%)	19 (18%)
Infant Feeding Method*		
EBF	71 (60%)	64 (62%)
Mostly breastfed	23 (19%)	20 (19%)
Mixed feeding	8 (7%)	7 (7%)
Commercial milk formula only	16 (14%)	13 (13%)

*EBF (no other liquid or solid from any other source entered the infant’s mouth); Mostly *breastfed* (human milk being the infant’s predominant source of nourishment, the infant may have received water, water-based drinks (sweetened and flavored water, teas, infusions, etc.), fruit juice, oral rehydration salts solution, drop and syrup forms of vitamins, minerals, and medicines, and ritual fluids (in limited quantities), but no food-based fluid) [10]; *mixed feeding* (feeding an infant breastmilk and commercial-milk-formula); *formula feeding (*feeding an infant only commercial-milk-formula)

Of the participants exposed to IC violation (94%, *n* = 104), one in three received free gifts with visible branding (29%, 30/104) and one in three (27%, 28/104) reported seeing commercial milk formula promotional materials. Of those who reported seeing promotional materials, the following facilities were identified: doctor offices (79%, *n* = 22/28), supermarkets (75%, *n* = 21/28), drug stores (71%, *n* = 20/28), pharmacies (50%, *n* = 14/28), hospitals (43%, *n* = 12/28), public health clinics (32%, *n* = 9/28), and locations such as clothing stores (29%, *n* = 8/28).

There were no differences in socio-demographics or infant feeding choice between exposed (87%) to and those not exposed (13%) to any IC violation (e.g., promotions, receiving free samples) (*p* >.05, data not shown). For any variable,< 5% of data were missing.

## Discussion

The current study showed that the vast majority of mothers (87%) were exposed to at least one IC violation. This percentage is higher than that reported in a recent global study by Lutter et al., on eight countries that reported 64% of mothers were exposed to at least one IC violation [8]. A recently published global scoping review by Becker et al., systematically examined the published and gray literature and reported on the exposure of mothers to IC violations between 1981 and 2021. The authors reported that the majority of studies identified violations of the IC, which included point-of-sale marketing (i.e., physical and online retailers), mass media (i.e., TV, radio, and print), and branding advertised in health facilities, with new mothers as the primary target. The most frequently reported violations were receiving coupons, discount codes and free samples of commercial milk formula directly from the manufacturers and distributors. Promotional practices included direct contact by manufacturers with mothers through email, direct messaging and phone calls. Fewer participants reported receiving free samples from healthcare professionals or retailers [16].

The 2006–2007 Canadian Maternity Experiences Survey investigated mothers’ experiences with healthcare centers and reported one in three mothers were given or offered free formula samples by health care providers [10]. In the present study, most participants reported receiving free samples of commercial milk formula directly from the manufacturers (57%). While fewer study participants received samples from doctors (12%) or nurses (6%).Of the study participants exposed to IC violations, one in three participants reported viewing promotional material for commercial milk formula most frequently in doctor offices, followed by supermarkets, pharmacies and public health clinics. In the study by Lutter et al., the authors reported that 63% of retail stores and pharmacies had visible promotional material on display [8]. In our study, manufacturers and distributors of commercial milk formula failed to comply with the IC, particularly Articles 5 and 6. Most participants were contacted directly by commercial milk formula companies via email, text messaging, mail or telephone with marketing messages.

Based on the high prevalence of IC violations observed in the current and other published studies, it is not surprising that the marketing expenditures of the commercial milk formula industry measured in dollars has increased by more than 3000% since 1978 and is now estimated at $56 billion dollars [6]. In addition, advertising occurs in print and online in pharmacies and physicians’ offices, and free samples are distributed most often directly to consumers [17].

The WHO Baby-Friendly Initiative (BFI) has been implemented in many facilities in Canada and around the world and provides an integrated framework for best practices in healthcare facilities that support optimal maternal child health for mothers and babies. For a birthing facility to become a designated BFI facility by a WHO qualified assessor, and adherence to ten steps is required to protect, promote and support breastfeeding in the hospital and in the community. For example, ensuring mothers are supported in breastfeeding in the first hour after birth. A BFI hospital designation also requires adherence to the IC [18]. In Canada in 2021, there were 30 hospital/birthing centers and 112 community health services designated Baby-Friendly (i.e., 142 out of a possible 1200) [19]. In NL, one of eight birthing facilities is designated Baby-Friendly [20].

We did not include online exposure to IC violations in the current study survey; however, we do know that expectant mothers or birthing parents seek out information on the worldwide web where direct-to-consumer marketing of commercial milk formula takes place [21–23]. A recent US study explored manufacturer websites and examined five infant formula companies, including three of the biggest manufacturers (i.e., Similac, Enfamil, and Gerber). Screenshots were taken from the websites, revealing that 29% provided coupons, discounts or rewards related to commercial milk formula. Additionally, 44% mentioned the benefits of commercial milk formula, while only 26% mentioned the benefits of breastfeeding [23]. While the IC explicitly does not mention online marketing, these screenshots provide evidence for breaching Article 4.2 “Information and education”, which states that any visual marketing should clearly include information about the superiority and benefits of human milk and the risks of infant formula [1]. It is common for health and nutritional claims to appear on commercial milk formula products and promotional materials despite international guidance prohibiting this type of marketing [24]. For example, during the pandemic, there was an increase in IC violations through the advertising of ‘immune benefits’ of commercial milk formula [25]. The WHO recently completed a review of evidence that demonstrated the scope and impact of digital marketing strategies used in the promotion of commercial milk formula [26]. Although the IC covers advertising regardless of the medium, online advertising was a challenge not anticipated by the WHO in 1981, and future amendments will be required to monitor and regulate the developments in digital marketing.

### Limitations

In our study, recruitment was limited to a convenience sample in one region of Canada and the socioeconomic status of study participants was higher than the population it represented. Therefore, the findings may have limited external validity [11]. The use of internet communities, social media, and email for recruitment may lead to selection bias, as only those with access to technology or who are online can participate. As participants reflected on past exposures, recall bias may have occurred; however, the impact is likely an underreporting of violations rather than an overestimation of study results.

## Conclusions

The majority of participants in this study reported being exposed to at least one IC violation, including receiving free samples, discounts, vouchers and coupons for commercial milk formula. Participants were contacted directly by manufacturers of commercial milk formula via text messaging, email, mail or phone. Commercial milk formula companies violate the IC of Marketing of Breast-Milk Substitutes through the use of marketing, advertising, and promotional techniques aimed at mothers of young infants.

### Electronic supplementary material

Below is the link to the electronic supplementary material.

Supplementary Material 1: Description of articles included in the IC adapted for the study survey.

## Data Availability

Data for this article can be made available by request to the corresponding author.

## References

[1] World Health Organization. International code of marketing of breast-milk substitutes. 1981. Available from: https://breastfeedingcanada.ca/wp-content/uploads/2020/03/TheCode-En.pdf.

[2] World Health Organization. Marketing of breast-milk substitutes: national implementation of the International Code, Status report 2022a. Available from: https://www.who.int/publications/i/item/9789240048799.

[3] Rollins NC, Bhandari N, Hajeebhoy N, Horton S, Lutter CK, Martines JC (2016). Why invest, and what it will take to improve breastfeeding practices?. Lancet.

[4] Soldavini J, Taillie LS (2017). Recommendations for adopting the International Code of marketing of breast-milk substitutes into U.S. policy. J Hum Lact.

[5] Victora CG, Bahl R, Barros AJ, França GV, Horton S, Krasevec J (2016). Breastfeeding in the 21st century: Epidemiology, mechanisms, and lifelong effect. Lancet.

[6] Baker P, Russ K, Kang M, Santos TM, Neves P, Smith J (2021). Globalization, first-foods systems transformations and corporate power: a synthesis of literature and data on the market and political practices of the transnational baby food industry. Globalization Health.

[7] World Health Organization. How the marketing of formula milk influences our decisions on infant feeding. Report. 2022. Available from: https://www.who.int/publications/i/item/9789240044609.

[8] Lutter CK, Hernández-Cordero S, Grummer-Strawn L, Lara-Mejía V, Lozada-Tequeanes AL (2022). Violations of the International Code of marketing of breast-milk substitutes: a multicountry analysis. BMC Public Health.

[9] Levitt C, Hanvey L, Kaczorowski J, Chalmers B, Heaman M, Bartholomew S (2011). Breastfeeding policies and practices in Canadian hospitals: comparing 1993 with 2007. Birth.

[10] Public Health Agency of Canada. What mothers say: The Canadian maternity experiences survey. 2009. Available from: https://www.canada.ca/content/dam/phac-aspc/migration/phac-aspc/rhs-ssg/pdf/survey-eng.pdf.

[11] Statistics Canada, Census, Profile. 2021 Census of Population. 2021. Available from: Profile table, Census Profile, 2021 Census of Population - Newfoundland and Labrador [Province]. https://www.statcan.gc.ca/.

[12] St Croix KA (2021). Supporting breastfeeding in rural Newfoundland and Labrador communities during COVID-19. Can J Public Health.

[13] Public Health Agency of Canada. Canada’s Breastfeeding Progress Report. 2022. Available from: https://health-infobase.canada.ca/breastfeeding/.

[14] Labbok MH, Starling A (2012). Definitions of breastfeeding: call for the development and use of consistent definitions in research and peer-reviewed literature. Breastfeed Med.

[15] International Baby Food Action Network (IBFAN). Code Monitoring Kit. 2019. Available from: https://www.babymilkaction.org/wp-content/uploads/2021/04/2019-CMK-Final.pdf.

[16] Becker GE, Zambrano P, Ching C, Cashin J, Burns A, Policarpo E (2022). Global evidence of persistent violations of the International Code of marketing of breast-milk substitutes: a systematic scoping review. Matern Child Nutr.

[17] Piwoz EG, Huffman SL (2015). The impact of marketing of breast-milk substitutes on WHO-recommended breastfeeding practices. Food Nutr Bull.

[18] World Health Organization. Implementation guidance: Protecting, promoting and supporting breastfeeding in facilities providing maternity and newborn services. The revised Baby-friendly Hospital Initiative. 2018. Available from: WHO UNICEF-bfhi-implementation-2018-En.pdf (breastfeedingcanada.ca).

[19] Statista. Number of hospital establishments in Canada as of 2020, by province. 2021. Available from: https://www.statista.com/statistics/440923/total-number-of-hospital-establishments-in-canada-by-province/.

[20] Breastfeeding Committee for Canada. Baby-Friendly Facilities in Canada. 2021. Available from: https://breastfeedingcanada.ca/wp-content/uploads/2021/01/2021-January-Designated-Facilities-in-Canada-en.pdf.

[21] Taştekin Ouyaba A, İnfal Kesim S (2021). The effect of the internet on decision-making during pregnancy: a systematic review. Arch Womens Ment Health.

[22] Abrahams SW (2012). Milk and social media: Online communities and the International Code of marketing of breast-milk substitutes. J Hum Lact.

[23] Pomeranz JL, Chu X, Groza O, Cohodes M, Harris JL. Breastmilk or infant formula? Content analysis of infant feeding advice on breastmilk substitute manufacturer websites. Public Health Nutr. 2021;1–9. 10.1017/S1368980021003451 PMC10346044 34517933

[24] Cheung KY, Petrou L, Helfer B, Porubayeva E, Dolgikh E, Ali S, et al. Health and nutrition claims for infant formula: International cross-sectional survey. BMJ. 2023;380. 10.1136/bmj-2022-071075. 10.1136/bmj-2022-071075 PMC9930154 36792145

[25] Ching ZP, Nguyen TT, Tharaney M, Zafimanjaka MG, Mathisen R (2021). Old tricks, new opportunities: how companies violate the International Code of marketing of breast-milk substitutes and undermine maternal and child health during the Covid-19 pandemic. Int J Environ Res Public Health.

[26] World Health Organization. Launch of new WHO report on the scope and impact of digital marketing for the promotion of breast-milk substitutes. 2022b. Available from: https://www.who.int/news-room/events/detail/2022/04/29/default-calendar/launch-of-new-who-report-on-the-scope-and-impact-of-digital-marketing-for-the-promotion-of-breast-milk-substitutes.

