# Supplementary material for: High rates of International Code violations: a cross-sectional study in a region of Canada with low breastfeeding rates
Source: BMC Res Notes. 2024 Mar 12;17:71. doi: 10.1186/s13104-024-06725-8 (PMC10935955; doi:10.1186/s13104-024-06725-8)
Supplement: Supplementary file 1 — Supplementary Material 1: Description of articles included in the IC adapted for the study survey. [file 13104_2024_6725_MOESM1_ESM.docx]

Supplementary Table

| Description of Articles 5-11 of the International Code* | |
| --- | --- |
| Article number / Name | Article description |
| Article 5  *The General Public and Mothers* | Article 5.1 states “There should be no advertising or other form of promotion to the general public of products within the scope of this Code” (WHO, 1981, p.10).  Article 5.2 states “Manufacturers and distributors should not provide, directly or indirectly, to pregnant women, mothers or members of their families, samples of products within the scope of this Code” (WHO, 1981, p.10).  Article 5.3 states “In conformity with paragraphs 1 and 2 of this Article, there should be no point-of-sale advertising, giving of samples, or any other promotion device to induce sales directly to the consumer at the retail level, such as special displays, discount coupons, premiums, special sales, loss-leaders and tie-in sales, for products within the scope of this Code. This provision should not restrict the establishment of pricing policies and practices intended to provide products at lower prices on a long-term basis” (WHO, 1981, p.11).  Article 5.4 states “Manufacturers and distributors should not distribute to pregnant women or mothers or infants and young children any gifts of articles or utensils which may promote the use of breast-milk substitutes or bottle-feeding” (WHO, 1981, p.11).  Article 5.5 states “Marketing personnel, in their business capacity, should not seek direct or indirect contact of any kind with pregnant women or with mothers of infants and young children” (WHO, 1981, p.11). |
| Article number / Name | Article description |
| Article 6  *Health Care Systems* | Article 6.1 states “The health authorities in Member States should take appropriate measures to encourage and protect breast-feeding and promote the principles of this Code, and should give appropriate information and advice to health workers in regard to their responsibilities, including the information specified in Article 4.2.” (WHO, 1981, p.11).  Article 6.2 states “No facility of a health care system should be used for the purpose of promoting infant formula (IF) or other products within the scope of this Code. This Code does not, however, preclude the dissemination of information to health professionals as provided in Article 7.2.” (WHO, 1981, p.11).  Article 6.3 states “Facilities of health care systems should not be used for the display of products within the scope of this Code, for placards or posters concerning such products, or for the distribution of material provided by a manufacturer or distributor other than that specific to Article 4.3.” (WHO, 1981, p.11).  Article 6.4 states “The use by the health care system of "professional service representatives", "mothercraft nurses" or similar personnel, provided or paid for by manufacturers or distributors, should not be permitted. 6.5 Feeding with infant formula, whether manufactured or home-prepared, should be demonstrated only by health workers, or other community workers if necessary; and only to the mothers or family members who need to use it; and the information given should include a  clear explanation of the hazards of improper use” (WHO, 1981, p.11).  Article 6.6 states “Donations or low-price sales to institutions or organizations of supplies of infant formula or other products within the scope of this Code, whether for use in the institutions or for distribution outside them, may be made. Such supplies should only be used or distributed for infants who have to be fed on breast-milk substitutes. If these supplies are distributed for use outside the institutions, this should be done only by the institutions or organizations concerned. Such donations or low-price sales should not be used by manufacturers or distributors as a sales inducement” (WHO, 1981, p.11).  Article 6.7 states “Where donated supplies of infant formula or other products within the scope of this Code are distributed outside an institution, the institution or organization should take steps to ensure that supplies can be continued as long as the infants concerned need them. Donors, as well as institutions or organizations concerned, should bear in mind this responsibility” (WHO, 1981, p.12).  Article 6.8 states “Equipment and materials, in addition to those referred to in Article 4.3, donated to a health care system may bear a company's name or logo, but should not refer to any proprietary product within the scope of this Code” (WHO, 1981, p.12). |
| Article number / Name | Article description |
| Article 7  *Health Care Workers* | Article 7.1 states “Health workers should encourage and protect breast-feeding; and those who are concerned in particular with maternal and infant nutrition should make themselves familiar with their responsibilities under this Code, including the information specified in Article 4.2” (WHO, 1981, p.12).  Article 7.2 states ``Information provided by manufacturers and distributors to health professionals regarding products within the scope of this Code should be restricted to scientific and factual matters, and such information should not imply or create a belief that bottlefeeding is equivalent or superior to breast-feeding. It should also include the information specified in Article 4.2” (WHO, 1981, p.12).  Article 7.3 states “No financial or material inducements to promote products within the scope of this Code should be offered by manufacturers or distributors to health workers or members of their families, nor should these be accepted by health workers or members of their families” (WHO, 1981, p.12).  Article 7.4 states “Samples of infant formula or other products within the scope of this Code, or of equipment or utensils for their preparation or use, should not be provided to health workers except when necessary for the purpose of professional evaluation or research at the institutional level. Health workers should not give samples of infant formula to pregnant women, mothers of infants and young children, or members of their families” (WHO, 1981, p.12).  Article 7.5 states “Manufacturers and distributors of products within the scope of this Code should disclose to the institution to which a recipient health worker is affiliated any contribution made to him or on his behalf for fellowships, study tours, research grants, attendance at professional conferences, or the like. Similar disclosures should be made by the recipient” (WHO, 1981, p.12). |
| Article number / Name | Article description |
| Article 8  *Persons employed by manufacturers and distributors* | Article 8.1 states “In systems of sales incentives for marketing personnel, the volume of sales of products within the scope of this Code should not be included in the calculation of bonuses, nor should quotas be set specifically for sales of these products. This should not be understood to prevent the payment of bonuses based on the overall sales by a company of other products marketed by it” (WHO, 1981, p.12-13).  Article 8.2 states “Personnel employed in marketing products within the scope of this Code should not, as part of their job responsibilities, perform educational functions in relation to pregnant women or mothers of infants and young children. This should not be understood as preventing such personnel from being used for other functions by the health care system at the request and with the written approval of the appropriate authority of the government concerned” (WHO, 1981, p.13). |
| Article number/Name  Article 9  *Labelling* | Article 9.1 states “Labels should be designed to provide the necessary information about the appropriate use of the product, and so as not to discourage breast-feeding” (WHO, 1981, p.13).  Article 9.2 states “Manufacturers and distributors of infant formula should ensure that each container as a clear, conspicuous, and easily readable and understandable message printed on it, or on a label which cannot readily become separated from it, in an appropriate language, which includes all the following points: (a) the words "Important Notice" or their equivalent; (b) a statement of the superiority of breastfeeding; (c) a statement that the product should be used only on the advice of a health worker as to the need for its use and the proper method of use; (d) instructions for appropriate preparation, and a warning against the health hazards of inappropriate preparation. Neither the container nor the label should have pictures of infants, nor should they have other pictures or text which may idealize the use of infant formula. They may, however, have graphics for easy identification of the product as a breastmilk substitute and for illustrating methods of preparation. The terms "humanized", "materialized" or similar terms should not be used. Inserts giving additional information about the product and its proper use, subject to the above conditions, may be included in the package or retail unit. When labels give instructions for modifying a product into infant formula, the above should apply” (WHO, 1981, p.13).  Article 9.3 states “Food products within the scope of this Code, marketed for infant feeding, which do not meet all the requirements of an infant formula, but which can be modified to do so, should carry on the label a warning that the unmodified product should not be the sole source of nourishment of an infant. Since sweetened condensed milk is not suitable for infant feeding, nor for use as a main ingredient of infant formula, its label should not contain purported instructions on how to modify it for that purpose” (WHO, 1981, p.13).  Article 9.4 states “The label of food products within the scope of this Code should also state all the following points: (a) the ingredients used; (b) the composition/analysis of the product; (c) the storage conditions required; and (d) the batch number and the date before which the product is to be consumed, taking into account the climatic and storage conditions of the country concerned” (WHO, 1981, p.13). |
| Article number / Name | Article description |
| Article 10 *Quality* | Article 10.1 states “The quality of products is an essential element for the protection of the health of infants and therefore should be of a high recognized standard” (WHO, 1981, p.14).  Article 10.2 states “Food products within the scope of this Code should, when sold or otherwise distributed, meet applicable standards recommended by the Codex Alimentarius Commission and also the Codex Code of Hygienic Practice for Foods for Infants and Children” (WHO, 1981, p.14). |
| Article number/ Name  Article 11  *Implementation and Monitoring* | Article 11.1 states “Governments should take action to give effect to the principles and aim of this Code, as appropriate to their social and legislative framework, including the adoption of national legislation, regulations or other suitable measures. For this purpose, governments should seek, when necessary, the cooperation of WHO, UNICEF and other agencies of the United Nations system. National policies and measures, including laws and regulations, which are adopted to give effect to the principles and aim of this Code should be publicly stated, and should apply on the same basis to all those involved in the manufacture and marketing of products within the scope of this Code” (WHO, 1981, p.14).  Article 11.2 states “Monitoring the application of this Code lies with governments acting individually, and collectively through the World Health Organization as provided in paragraphs 6 and 7 of this Article. The manufacturers and distributors of products within the scope of this Code, and appropriate nongovernmental organizations, professional groups, and consumer organizations should collaborate with governments to this end” (WHO, 1981, p.14).  Article 11.3 states “Independently of any other measures taken for implementation of this Code, manufacturers and distributors of products within the scope of this Code should regard themselves as responsible for monitoring their marketing practices according to the principles and aim of this Code, and for taking steps to ensure that their conduct at every level conforms to them” (WHO, 1981, p.14).  Article 11.4 states “Nongovernmental organizations, professional groups, institutions and individuals concerned should have the responsibility of drawing the attention of manufacturers or distributors to activities which are incompatible with the principles and aim of this Code, so that appropriate action can be taken. The appropriate governmental authority should also be informed” (WHO, 1981, p.14).  Article 11.5 states “Manufacturers and primary distributors of products within the scope of this Code should apprise each member of their marketing personnel of the Code and of their responsibilities under it” (WHO, 1981, p.14).  Article 11.6 states “In accordance with Article 62 of the Constitution of the World Health Organization, Member States shall communicate annually to the Director-General information on action taken to give effect to the principles and aim of this Code” (WHO, 1981, p.14).  Article 11.7 states “The Director-General shall report in even years to the World Health Assembly on the status of implementation of the Code; and shall, on request, provide technical support to Member States preparing national legislation or regulations, or taking other appropriate measures in implementation and furtherance of the principles and aim of this Code” (WHO, 1981, p.15). |
| Digital Marketing | *The draft [Decision](https://apps.who.int/gb/ebwha/pdf_files/EB150/B150_23-en.pdf) approved by the January Executive Board meeting (EB150/7) was adopted without amendment. It calls on the WHO Director General to *“develop guidance for Member States on regulatory measures aimed at restricting the digital marketing of breastmilk substitutes, so as to ensure that existing and new regulations designed to implement the International Code of Marketing Breastmilk Substitutes and relevant Health Assembly resolutions subsequent to its adoption adequately address digital marketing practices..”* and to report back in 2024. Any marketing and promotion whether digital or otherwise is in violation of the International Code and subsequent WHA resolutions. The decision is for countries to develop regulatory measures to restrict this form of Code violations. |

*adopted in 1981, more provisions have been adopted in subsequent resolutions.
